# Generative AI-Assisted Academic Writing Experience and University Students’ Academic Self-Efficacy: Parallel Indirect Associations Through Academic Emotions

**DOI:** 10.3390/jintelligence14090221

**Published:** 2026-09-15

**Authors:** Tingzhi Han, Yiwen Yuan, Nitong Zhou, Zijing Fan

**Affiliations:** School of Humanities, Jiangnan University, Wuxi 214122, China

**Keywords:** generative artificial intelligence, academic writing, academic emotions, academic self-efficacy, statistical suppression, human–AI interaction

## Abstract

Generative artificial intelligence (GenAI) is increasingly used in academic writing, yet its associations with students’ broader academic emotions and capability beliefs remain unclear. Drawing on control–value theory and social cognitive theory, this cross-sectional study examined positive and negative academic emotions as parallel statistical pathways between GenAI-assisted writing experience and general academic self-efficacy. Participants were 1128 students from universities in eastern China. A covariate-adjusted parallel pathway model was estimated with 5000 bootstrap resamples. GenAI-assisted writing experience was positively associated with self-efficacy (total association B = 0.733, β = 0.670) and broader positive emotions (B = 0.778, β = 0.631), and negatively associated with broader negative emotions (B = −0.457, β = −0.298). The positive-emotion indirect association was 0.471 (standardized β = 0.431), 95% CI [0.409, 0.531], whereas the negative-emotion indirect association was 0.033 (standardized β = 0.030), 95% CI [0.018, 0.052]. The direct association remained positive (B = 0.229, β = 0.209). After positive academic emotions were controlled, the coefficient linking GenAI-writing experience with negative emotions changed from negative to positive, indicating a statistical suppression pattern. A sensitivity model excluding emotional-motivation items reproduced both indirect associations. Given the limited discriminant validity between positive emotions and self-efficacy and the concurrent self-report design, the larger positive indirect association warrants cautious interpretation. The findings indicate that GenAI-assisted writing experience is associated with broader academic emotions and efficacy beliefs while retaining a smaller residual negative-emotion component.

## 1. Introduction

Generative artificial intelligence (AI) is rapidly becoming a routine component of university writing. Students use large language models to brainstorm, organize arguments, revise language, and obtain immediate feedback, creating iterative human–AI exchanges that can personalize support while reshaping agency, evaluation, and authorship. Recent research shows that GenAI-assisted academic writing involves recurring cycles of planning, monitoring, feedback seeking, and revision rather than a single episode of tool use (Jia et al., 2026). At the same time, cognitive offloading to GenAI may weaken perceived ownership of the work, and dependence on generated content may reduce critical engagement (J. Su et al., 2026; Yan et al., 2024). These competing possibilities make students’ affective and capability-related responses important for understanding responsible AI-supported learning.

Academic self-efficacy refers to beliefs about one’s capability to complete academic tasks (Bandura, 1997) and predicts engagement, persistence, and achievement (Zimmerman, 2000). It is domain- and level-specific: confidence in coordinating prompts, evaluating outputs, and revising with AI is not identical to confidence in learning, managing study demands, and succeeding across academic contexts. Academic self-efficacy is not an intelligence measure; rather, it is a motivational belief that influences whether students initiate, sustain, and regulate cognitive effort when confronting demanding tasks. Examining it in GenAI-supported writing therefore helps distinguish perceived capability from cognitive ability while clarifying how technology-mediated experiences may shape the deployment of cognitive resources. In these environments, efficacy beliefs may strengthen through perceived progress and feedback but may also coexist with anxiety, stress, or uncertainty about dependence and authorship (B. Zhang et al., 2026).

Recent research has examined behavioral regulation during GenAI-assisted writing (Jia et al., 2026), emotional responses during AI-assisted learning (Hoang, 2026), links among AI support, anxiety, stress, and self-efficacy (B. Zhang et al., 2026), and the relations of AI-supported learning with critical thinking and creativity (Yang et al., 2026). Two gaps remain. First, existing research often focuses on task-specific emotions or AI-use efficacy, leaving it unclear whether writing-related AI experiences are associated with students’ broader academic emotions and general beliefs about academic capability. Second, positive and negative emotions are often examined separately, which can conceal their unequal contributions and possible statistical suppression. Integrating Control–Value Theory (CVT; Pekrun, 2006, 2024) and Social Cognitive Theory (SCT; Bandura, 1997), this study tests positive and negative academic emotions as parallel statistical pathways between AI-assisted academic writing experience and general academic self-efficacy. Rather than treating efficacy as a proxy for intelligence, the analysis positions it as a belief about mobilizing academic capabilities. The study contributes a large, multidisciplinary Chinese higher-education sample, distinguishes tool-related experience from broader affect and capability beliefs, and evaluates whether a generally favorable AI-writing experience can retain a smaller adverse emotional correlate.

## 2. Theoretical Background and Hypothesis Development

### 2.1. AI-Assisted Academic Writing and Academic Self-Efficacy

AI-assisted academic writing can be understood as a technology-mediated cognitive and self-regulatory experience spanning planning, drafting, feedback, revision, perceived effectiveness, emotional motivation, and ethical decision-making (Khojasteh et al., 2025). Repeated exchanges with a generative system can reduce task ambiguity, distribute cognitive load, clarify requirements, and provide rapid examples or feedback (Escalante et al., 2023; Y. Su et al., 2023). Behavioral evidence indicates that learners coordinate multiple self-regulatory actions while writing with GenAI, although poorly coordinated patterns may signal overreliance or shallow engagement (Jia et al., 2026). Complementing this process-focused evidence, Mekheimer (2025) reported higher post-test writing scores—particularly for organization and language use—in a Grammarly-supported feedback group than in a teacher-feedback group, together with positive within-group associations between limited generative-feature use and revision frequency. Interview participants also described reduced frustration and greater confidence during revision, although these qualitative perceptions should not be equated with general academic self-efficacy. If students outsource evaluation or reasoning, the same affordances can encourage cognitive offloading and weaken ownership of the resulting work (J. Su et al., 2026).

Repeated successful completion of writing subtasks may provide mastery-like information, while generated examples and feedback can make difficult work appear manageable. However, externally supported success is not equivalent to independently demonstrated ability: reliance on generation can weaken ownership and confidence calibration, and trust in a system need not increase confidence as an autonomous writer (Dhillon et al., 2024; Park et al., 2026; J. Su et al., 2026). Ambiguous rules may also create ethical pressure (Barrett & Pack, 2023; Lund et al., 2023). The expected association is therefore positive on average but theoretically bounded by how actively and critically students direct, evaluate, and revise AI output.

This distinction is especially important in human–AI collaboration. Task-specific AI-writing self-efficacy includes process regulation, language support, and ethical-integrity judgments (Yao & Fan, 2026b), whereas general academic self-efficacy spans learning ability and learning behavior. Self-efficacy concerns perceived capability, while intelligence refers to cognitive capacities. The present study therefore asks whether experience in a specific human–AI activity is associated with a broader belief about academic functioning; it does not test whether GenAI use changes intelligence.

Accordingly, supportive AI-writing experiences may supply efficacy-relevant information, whereas overdelegation or unclear norms may constrain that benefit.

Based on the above theoretical reasoning and empirical evidence, we propose:

**H1.**  *AI-assisted academic writing experience is positively associated with university students’ academic self-efficacy.*

### 2.2. Positive Academic Emotions as an Indirect Pathway

CVT defines achievement emotions as affective experiences tied to achievement activities or outcomes and explains them through appraisals of control and value (Pekrun, 2006, 2024). AI can increase perceived control by reducing ambiguity, accelerating feedback, and making progress visible. When students value a writing task and feel able to influence its outcome, enjoyment, hope, and pride are more likely. Positive emotions can broaden attention, sustain exploration, and build psychological resources (Fredrickson, 2001), while SCT treats affective states as efficacy-relevant information. From a cognitive-resource perspective, constructive emotions may support sustained attention and strategic engagement without implying higher cognitive ability. Evidence that enjoyment links large-language-model acceptance with writing self-efficacy further supports this pathway (Sun et al., 2026).

**H2a.**  *AI-assisted academic writing experience is indirectly associated with academic self-efficacy through broader positive academic emotions.*

### 2.3. Negative Academic Emotions as an Indirect Pathway

Academic writing is cognitively demanding and evaluative, often co-occurring with anxiety, hopelessness, and boredom. Generative AI may be associated with lower levels of these broader academic emotions by offering immediate guidance and reducing uncertainty. Because negative arousal can consume task-relevant attention and undermine efficacy judgments (Bandura, 1997), lower negative emotion may accompany stronger general self-efficacy. Yet inaccurate output, cognitive offloading, dependence, originality concerns, and unclear authorship boundaries can create new tension (J. Su et al., 2026; Wang & Fussell, 2026; L. Zhang & Xu, 2025). Critical AI literacy—understanding, strategically applying, evaluating, and ethically regulating AI—may determine whether support becomes reassurance or uncertainty (Yao & Fan, 2026a). Because the emotion scale retained its general classroom and learning frames, the proposed pathway is interpreted as a cross-context association rather than an AI-specific emotional process.

Thus, negative emotions may carry a smaller and less uniform indirect association than positive emotions.

**H2b.**  *AI-assisted academic writing experience is indirectly associated with academic self-efficacy through broader negative academic emotions.*

### 2.4. Differential Pathway Strength and the Suppression Pattern

Positive and negative pathways may differ in magnitude. AI’s immediate feedback can rapidly create appraisals of progress, enjoyment, and accomplishment, whereas associations with anxiety or hopelessness may depend more strongly on prior writing competence, institutional rules, and evaluative skill. Research has similarly found enjoyment to be a stronger statistical pathway than anxiety between large-language-model acceptance and writing self-efficacy (Sun et al., 2026). Comparing the two pathways can therefore show whether favorable and unfavorable affect contribute equally to the observed association, while remaining agnostic about temporal order.

**H2c.**  *The indirect association through positive academic emotions is significantly larger than the indirect association through negative academic emotions.*

The zero-order association may also conceal statistically competing covariance components. AI can reduce perceived difficulty while simultaneously raising concerns about accuracy, dependence, originality, or authorship. If positive emotions share substantial variance with both AI experience and efficacy, controlling them may reveal a smaller residual association between AI experience and negative emotions. We therefore examine this possible suppression pattern exploratorily. Suppression is an algebraic property of the fitted regression model; it is not, by itself, evidence of a hidden psychological mechanism or of AI-caused distress.

RQ1. After controlling for positive academic emotions, does a statistical suppression pattern emerge between AI-assisted writing experience and negative academic emotions?

**Figure 1:**
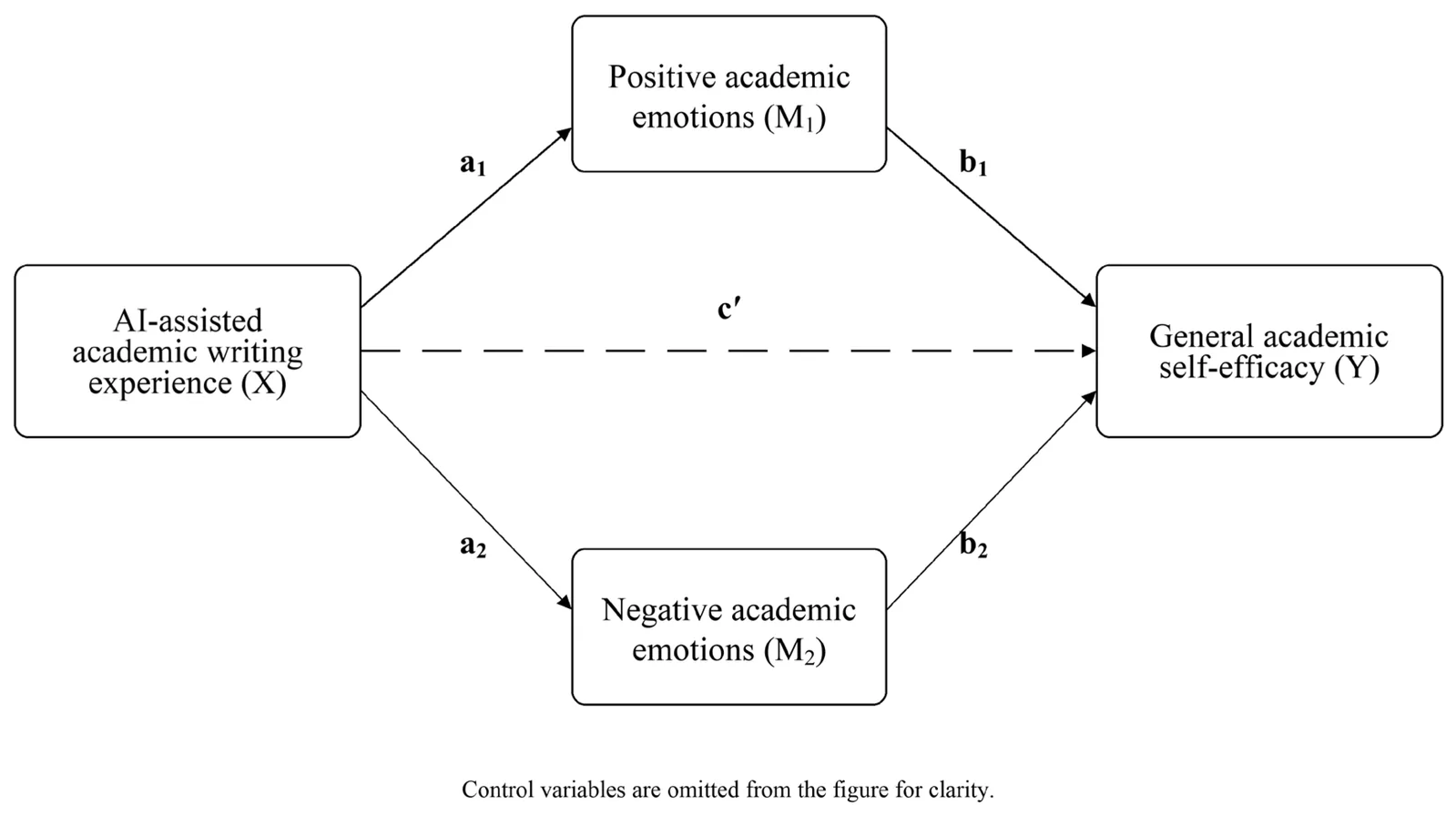
Conceptual model of the parallel pathways linking AI-assisted academic writing experience and general academic self-efficacy. X = AI-assisted academic writing experience; M_1_ = positive academic emotions; M_2_ = negative academic emotions; Y = general academic self-efficacy. The dashed arrow represents the direct path (c′); solid arrows represent paths through M_1_ (a_1_ and b_1_) and M_2_ (a_2_ and b_2_). Control variables—academic level, university type, AI-use frequency, and academic rank—are omitted for clarity.

The conceptual model incorporating all hypothesized paths is depicted in Figure 1.

## 3. Materials and Methods

### 3.1. Participants

A cross-sectional online survey was distributed through the Wenjuanxing platform to undergraduate and graduate students at universities in eastern China. Data were collected from 19 March to 14 April 2026 using convenience and snowball sampling. Eligibility required enrollment at a Chinese university, age 18 or older, and prior use of generative AI in academic writing. Before entering the questionnaire, participants read an electronic informed-consent statement describing the study purpose, voluntary participation, confidentiality, and the right to withdraw, and indicated their consent electronically. The study was conducted in accordance with the Declaration of Helsinki and ethical guidelines for educational research. Prospective ethical approval was obtained from the Ethics Committee of East China Normal University (IRB00013174; 27 February 2022). This approval explicitly covered the present research protocol and remained valid during data collection from 19 March to 14 April 2026.

Of 1278 submitted questionnaires, 150 were removed during platform and prespecified response-quality screening, based on completion in under 300 s, an incorrect response to the instructed-response item, or invariant responding across the substantive items. The retained dataset contained no completion times below 300 s, no failures on the instructed-response item, and no invariant responding across all substantive items. Criterion-specific counts for the 150 exclusions were not retained separately and therefore cannot be reconstructed. The analytic sample comprised 1128 complete cases (88.3% valid), with no missing focal or covariate values. It included 526 men (46.6%) and 602 women (53.4%); 549 students attended Double First-Class universities, and 579 attended other institutions. Table 1 provides the full profile.

### 3.2. Variables and Measures

The focal item scales used five response points from 1 (strongly disagree/completely inconsistent) to 5 (strongly agree/completely consistent). AI-use frequency was originally coded from 1 (never) to 5 (always); because eligibility required prior AI-writing use, the observed responses ranged from 2 (rarely) to 5 (always). Table 2 summarizes the instruments and measurement properties.

#### 3.2.1. AI-Assisted Academic Writing Experience

AI-assisted academic writing experience was measured with the 30-item AI-AWQ (Khojasteh et al., 2025), covering writing process, feedback improvement, perceived effectiveness, emotional motivation, and ethical considerations. The English instrument was translated into Chinese, back-translated, compared with the source, and contextually adapted for Chinese universities. In an exploratory, sample-specific refinement, two reverse-worded items with corrected item-total correlations below 0.30 were removed using the same N = 1128 sample used for subsequent model estimation: a technical-problems item (−0.22) and an originality-concern item (−0.10). Their removal improved perceived-effectiveness alpha from 0.577 to 0.743 and emotional-motivation alpha from 0.481 to 0.694. Because the item-removal decision and fit assessment were not independent, the reduced form is not presented as a newly validated scale. The removal of the originality item is treated as narrowed construct coverage rather than evidence that originality concerns are unimportant.

The resulting sample-specific reduced 28-item form showed acceptable five-factor fit, χ^2^(340) = 1270.07, CFI = 0.920, TLI = 0.911, RMSEA = 0.049, with item-level standardized loadings of 0.521–0.745. Subscale alphas ranged from 0.694 to 0.844. As a post hoc internal-stability check, the prespecified 28-item structure was fitted in two random halves (n = 564 each; seed = 20,260,726): half 1, χ^2^(340) = 842.25, CFI = 0.918, TLI = 0.909, RMSEA = 0.051; half 2, χ^2^(340) = 860.55, CFI = 0.906, TLI = 0.896, RMSEA = 0.052. This split-half check does not constitute independent external validation because item deletion was informed by the full sample. The 28 retained items were averaged to form the overall composite used in the pathway analyses. In the parcel-level four-construct model, the ethics parcel loaded weakly (0.304), whereas the other four parcels loaded 0.838–0.909; the score is therefore treated as a broad multidimensional composite rather than a strictly unidimensional reflective scale.

#### 3.2.2. Academic Emotions

Broader academic emotions were assessed with the 64-item class- and learning-related AEQ-S (Bieleke et al., 2021). The original classroom and learning frames were retained rather than rewritten for AI-assisted writing; for example, items referred to enjoying class and feeling hopeful about learning. Enjoyment, hope, and pride formed positive emotions (alpha = 0.952); anger, anxiety, shame, hopelessness, and boredom formed negative emotions (alpha = 0.973). Dimension means served as parcel indicators. Positive-emotion loadings were 0.906–0.950 (AVE = 0.857; CR = 0.947), and negative-emotion loadings were 0.788–0.933 (AVE = 0.769; CR = 0.943).

#### 3.2.3. Academic Self-Efficacy

General academic self-efficacy was measured with Liang’s (2000) 22-item Chinese scale. This instrument was selected because it was developed for Chinese university students, was available in the participants’ primary language, and assesses the broad learning-ability and learning-behavior beliefs required by the present research question rather than AI-writing-specific confidence. The original general academic wording was retained; no items were reframed for AI-assisted writing. Items 1–11 assess learning-ability efficacy and items 12–22 assess learning-behavior efficacy. The scale does not directly assess prompt design, output evaluation, or ethical AI use and is therefore interpreted as a broad academic belief. The sole reverse-worded item (item 20) was reverse-coded according to the original scoring instructions. Alpha was 0.915. To preserve the confirmed two-domain structure, two balanced parcels were formed within each domain (four parcels total); loadings were 0.800–0.871 (AVE = 0.713; CR = 0.909). The 22-item mean was used in pathway analysis.

#### 3.2.4. Control Variables

Academic level, university type, AI-use frequency, and self-reported academic rank were covariates. In the primary model, academic level and AI-use frequency were entered as ordered numeric scores, university type was binary (Double First-Class vs. other regular undergraduate universities), and academic rank was represented by four dummy variables with the top 20% group as the reference. Academic rank categories were top 20%, 20–40%, 40–60%, 60–80%, and bottom 20%. A sensitivity model dummy-coded academic level and AI-use frequency and additionally controlled for gender and broad discipline; its results are reported in the Supplementary Materials.

### 3.3. Data Analysis

Analyses used Python 3.9.6, semopy 2.3.11 with maximum-likelihood Wishart estimation for CFA, and statsmodels 0.14.6 for regression and case-bootstrap estimation. Measurement quality was evaluated through reliability, CFA, the Fornell–Larcker criterion (Fornell & Larcker, 1981), and the heterotrait–monotrait ratio (HTMT; Henseler et al., 2015), following general measurement guidance from Hair et al. (2019). The retained dataset contained no missing focal or covariate values, so all models used the same 1128 complete cases. HTMT intervals used 5000 case-bootstrap resamples with seed 20,260,720; the primary parallel pathway model used 5000 percentile case-bootstrap resamples with seed 20,260,721. Both unstandardized B and fully standardized β coefficients were calculated for focal paths, while bootstrap intervals remained on the unstandardized scale. The two indirect associations were contrasted directly. Because all focal variables were measured at one time point, pathway coefficients were interpreted as regression-based indirect associations rather than causal mediation. Sensitivity models using separately recorded seeds are reported in the Supplementary Materials.

For RQ1, negative academic emotions were regressed on AI-assisted writing experience, positive academic emotions, and the same covariates. A reversal in the coefficient for AI-writing experience relative to the model without positive emotions was interpreted as a statistical suppression pattern. This analysis was exploratory and was not interpreted causally.

Harman’s single-factor test was reported as a diagnostic rather than proof against common-method bias (Podsakoff et al., 2003). Reviewer-requested post hoc analyses included a full comparison of the four-factor and positive-emotion/self-efficacy merged models and a random split-half internal-stability check for the sample-specific reduced 28-item AI-AWQ form. Relative weight analysis (Johnson, 2000), no-covariate estimation, efficacy-domain sensitivity, the non-emotional AI-predictor model, and an enhanced dummy-coded covariate model were treated as sensitivity analyses because they extend rather than define the focal dual-path model.

## 4. Results

### 4.1. Common Method Bias and Validity Testing

The first unrotated factor accounted for 31.55% of variance, below the conventional 40% diagnostic threshold. This does not eliminate common-method risk, particularly because all focal variables were collected from the same respondents at one time. Using five AI subscale means, three positive-emotion dimensions, five negative-emotion dimensions, and four efficacy parcels, the four-factor measurement model fit acceptably according to CFI and TLI, χ^2^(113) = 945.03, CFI = 0.955, TLI = 0.946, although RMSEA = 0.081 was marginal. It clearly outperformed the one-factor model, χ^2^(119) = 6209.59, CFI = 0.673, TLI = 0.626, RMSEA = 0.213. Table 2 reports the estimates from this same final model.

The standardized latent correlation between positive emotions and self-efficacy was 0.942, compared with the square roots of the AVEs of 0.926 and 0.845, respectively (Table 3). The observed composite-score correlation was r = 0.877, and HTMT was 0.946, 95% bootstrap CI [0.920, 0.967], above the conventional 0.85 and 0.90 reference values (Table 4). An item-level HTMT sensitivity estimate was 0.926. The four-factor model, χ^2^(113) = 945.03, CFI = 0.955, TLI = 0.946, RMSEA = 0.081, fit better than a model merging positive emotions and self-efficacy, χ^2^(116) = 1152.33, CFI = 0.944, TLI = 0.935, RMSEA = 0.089; Δχ^2^(3) = 207.30, *p* < .001, and ΔCFI = −0.011 for the merged model. Taken together, the construct-level diagnostics indicate limited discriminant validity, whereas the significantly better fit of the four-factor model supports retaining the theoretically distinct constructs. Their relative pathway coefficients are therefore interpreted cautiously.

HTMT estimates for all other construct pairs were below 0.90. The positive-emotion–self-efficacy result was similar under balanced within-domain efficacy parceling and item-level estimation.

### 4.2. Descriptive Statistics and Correlations

Table 5 presents descriptive statistics and correlations. Absolute skewness was below 2 and absolute kurtosis below 7 (Kline, 2015). Using the sample-specific reduced 28-item AI-AWQ form, AI-writing experience correlated positively with positive emotions (r = 0.607) and self-efficacy (r = 0.643), and negatively with negative emotions (r = −0.329), all *p* < .001. Positive emotions and self-efficacy were strongly correlated (r = 0.877).

### 4.3. Parallel Pathway Analysis

**Figure 2:**
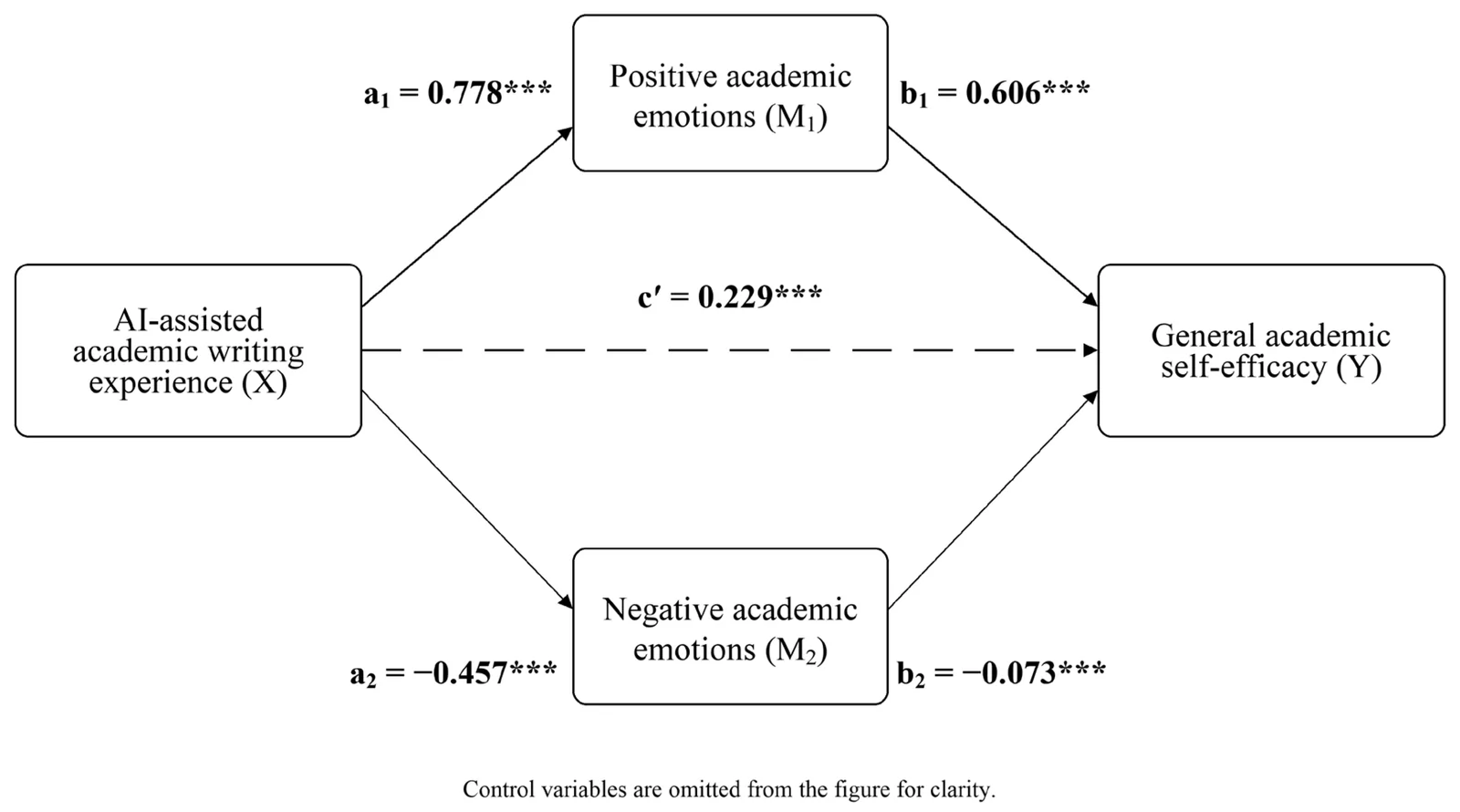
Results of the parallel pathway model. Unstandardized coefficients are reported; corresponding standardized coefficients are provided in Table 6. Control variables were included in the model but omitted from the figure for clarity. *** *p* < .001.

The parallel pathway model controlled for academic level, university type, AI-use frequency, and academic rank (Table 6; Figure 2). We use the term indirect association rather than mediation because all focal variables were measured concurrently. The coefficients do not establish temporal ordering, a causal process, a distinct psychological mechanism, or the absence of unmeasured confounding.

AI-writing experience was positively associated with general academic self-efficacy (total association B = 0.733, β = 0.670, *p* < .001), supporting H1. It was positively associated with broader positive emotions (a_1_: B = 0.778, β = 0.631, *p* < .001) and negatively associated with broader negative emotions (a_2_: B = −0.457, β = −0.298, *p* < .001). In the joint model, positive emotions were positively associated with efficacy (b_1_: B = 0.606, β = 0.683, *p* < .001), whereas negative emotions were negatively associated with efficacy (b_2_: B = −0.073, β = −0.102, *p* < .001). The direct association remained significant (c′: B = 0.229, β = 0.209, *p* < .001).

The positive-emotion indirect association was B = 0.471, β = 0.431, 95% CI [0.409, 0.531], and the negative-emotion indirect association was B = 0.033, β = 0.030, 95% CI [0.018, 0.052], supporting H2a and H2b, respectively. Their bootstrap contrast was B = 0.438, standardized contrast = 0.400, 95% CI [0.365, 0.506], supporting H2c. Expressed descriptively relative to the total unstandardized association, the two indirect coefficients represented 64.3% and 4.5%, respectively, and together represented 68.8%. These ratios describe the fitted coefficient decomposition and should not be interpreted as proportions of a causal effect.

Sensitivity models reproduced the pattern for learning-ability efficacy (positive indirect = 0.519, 95% CI [0.448, 0.589]; negative indirect = 0.035, 95% CI [0.016, 0.056]) and learning-behavior efficacy (positive indirect = 0.423, 95% CI [0.361, 0.484]; negative indirect = 0.032, 95% CI [0.016, 0.050]). To address content overlap from the AI-AWQ emotional-motivation dimension, a revised predictor was computed as the equal-weight mean of the 24 retained items from writing process, feedback improvement, perceived effectiveness, and ethical considerations, excluding all four emotional-motivation items. Both indirect associations remained significant (positive = 0.457, 95% CI [0.391, 0.517]; negative = 0.030, 95% CI [0.016, 0.047]) and the suppression pattern was preserved. This check reduces direct emotional-content overlap in the predictor but cannot eliminate common method variance or overlap among broader evaluative judgments. In a further enhanced model, dummy-coding academic level and AI-use frequency and adding gender and broad discipline produced similar estimates (positive indirect = 0.463, 95% CI [0.397, 0.526]; negative indirect = 0.032, 95% CI [0.017, 0.051]). Full sensitivity results are reported in the Supplementary Materials.

For RQ1, AI-writing experience was negatively associated with negative emotions before positive emotions were entered (B = −0.457) but positively associated with their residual variance after positive emotions were controlled (B = 0.194, *p* < .001). This sign reversal is a statistical suppression pattern: the coefficient for AI-writing experience changed after accounting for its strong shared association with positive emotions. It does not establish hidden AI-caused distress, identify dependence, cognitive offloading, originality, or authorship as its psychological source, or resolve temporal order. Those explanations were not directly measured and remain hypotheses for future research. Relative-weight and no-covariate analyses were consistent with the central statistical pattern and are reported in the Supplementary Materials.

## 5. Discussion

This study examined how AI-assisted academic writing experience was associated with broader academic emotions and general academic self-efficacy among 1128 students in Chinese higher education. Positive emotions carried the larger indirect association, negative emotions carried a smaller component, and a suppression pattern appeared after positive emotions were controlled. The results qualify an exclusively beneficial account of AI support, but their interpretation must remain noncausal and must account for the substantial empirical overlap between positive emotions and self-efficacy.

### 5.1. Theoretical Implications

First, the positive total association extends SCT to an AI-supported writing context in which generated examples, rapid feedback, and visible task progress may function as efficacy-relevant information. This complements evidence that GenAI support is related to psychological adaptation and self-efficacy (B. Zhang et al., 2026), while showing that the association extends beyond AI-specific confidence to a general belief about academic capability. Academic self-efficacy should not be equated with intelligence: it may influence whether students initiate, monitor, and persist in using cognitive resources, but the present results provide no evidence of a change in cognitive ability. The contribution is therefore about capability appraisal and cognitive agency within human–AI interaction.

Second, the substantially larger positive-emotion coefficient is consistent with CVT and SCT accounts in which appraisals of control and progress align with enjoyment, hope, pride, and efficacy judgments. Modeling positive and negative emotions together reveals their unequal statistical contributions, and the pattern persisted across both efficacy domains and after emotional-motivation items were removed from the AI-writing predictor. The larger positive-emotion coefficient should nevertheless be interpreted in light of the limited discriminant validity between positive emotions and self-efficacy and the concurrent self-report design. The significantly better fit of the four-factor model supports their theoretical separation, while future longitudinal and methodologically differentiated research is needed to clarify their temporal relationship. Accordingly, R^2^ = 0.799 should be understood as concurrent explained variance rather than causal explanatory power.

Third, the suppression result shows that favorable and unfavorable statistical correlates can coexist within the same reported AI-writing experience. The model does not reveal why the residual negative-emotion coefficient appeared. Factual reliability, dependence, cognitive offloading, originality, and authorship are plausible hypotheses, but none was directly measured as the source of the coefficient. The result is therefore a hypothesis-generating algebraic pattern in concurrent data, not confirmation of a dual psychological or causal mechanism.

Supplementary relative-weight results identified perceived effectiveness and emotional motivation as the two largest contributors across the three outcomes. The ethical-considerations dimension contributed little unique explained variance to the three outcomes in this sample. Given its weak parcel loading (0.304), limited item coverage, and the removal of an originality-related item, ethics-, authorship-, and originality-specific conclusions are more tentative than conclusions about writing process, feedback, or perceived effectiveness. The result should not be read as evidence that ethical considerations are unimportant; it instead supports direct measurement of ethical judgment, cognitive ownership, and responsible decision-making in future research.

### 5.2. Practical Implications

Given the cross-sectional design, the practical implications are design propositions to be tested rather than demonstrated intervention effects. In addition, recommendations concerning authorship, originality, and ethics are especially provisional because the ethical-considerations parcel was the weakest-loading and least well-represented component of the AI-writing composite. Three priorities nevertheless follow as testable directions from the observed pattern and its limitations.

First, GenAI-supported writing should preserve active cognitive agency. Learners can set goals before prompting, generate an initial position before consulting AI, compare human and AI drafts, justify revisions, and document how feedback changed their decisions. These activities keep planning, evaluation, and authorship with the learner and make fluency or speed insufficient evidence of learning. Instruction should also assess the reasoning behind a final text, not only the polished product.

Second, institutions should pair access with critical AI literacy and explicit interaction norms. Students need prompting skills, routines for verifying claims and sources, guidance on attribution and privacy, and strategies for deciding when AI assistance should be limited. Because AI-supported learning is also related to critical thinking and creativity (Yang et al., 2026), instruction should require students to challenge outputs, compare alternatives, and explain why an AI suggestion was accepted or rejected. These practices may help preserve independent judgment while retaining useful scaffolding.

Third, the smaller negative indirect association and suppression pattern justify monitoring, not assuming, emotional costs. Brief check-ins, reflective logs, and direct questions about dependence, originality, and authorship could test concerns not specifically measured by the present general emotion scale and weakened ethics component. Students who report low control or strong reliance may benefit from more human feedback and progressively reduced scaffolding, whereas more confident students may need tasks that require critique rather than acceptance of AI output. These proposals require experimental and longitudinal evaluation.

### 5.3. Limitations and Future Directions

Several limitations qualify the findings. First, cross-sectional self-report data cannot establish temporal ordering, causality, a distinct mechanism, or the absence of unmeasured confounding; efficacy may also shape AI use and emotion. Harman’s diagnostic and measurement-model comparisons do not remove common-method bias. Longitudinal, experimental, experience-sampling, behavioral, and multi-informant designs are needed. Institution identifiers and the exact number of participating universities were not retained, so within-university dependence could not be estimated, and standard errors could not be cluster-adjusted. If responses were positively correlated within universities, the reported standard errors and case-bootstrap confidence intervals may understate true sampling uncertainty. Future multisite studies should retain institution codes and use cluster-robust or multilevel estimation. In addition, criterion-specific counts for excluded questionnaires were not preserved, preventing retrospective reconstruction of overlap among screening rules. Project records document translation and back-translation of the AI-writing measure but do not retain translator qualifications or a separate cognitive-interview record.

Second, convenience sampling from eastern Chinese universities limits generalizability, and eligibility required prior AI-writing use. Future samples should include nonusers, central and western regions, other Asia-Pacific systems, disciplines, and levels of institutional AI readiness. Measurement invariance across these groups should be tested.

Third, deleting two reverse-worded AI-AWQ items was an exploratory, sample-specific refinement that improved reliability but narrowed coverage, particularly for originality concern. The post hoc split-half CFA suggests internal structural stability but is not independent validation because the full sample informed item removal. The trimmed 28-item form therefore requires replication and validation in an independent sample before being treated as a validated adaptation; future work should also assess originality anxiety separately. Fourth, general academic self-efficacy differs from AI-writing-specific efficacy; both should be measured to test whether task confidence transfers to broader beliefs.

Finally, future studies should use more differentiated wording, temporal separation, state-level emotion measures, and latent longitudinal models to clarify whether positive affect precedes capability beliefs or partly reflects them. Moreover, neither intelligence nor objective writing performance was measured. The findings therefore cannot show that GenAI changes cognitive ability, achievement, metacognitive calibration, or writing quality. Future studies should combine ability and performance measures with process traces, confidence judgments, direct ethics/authorship measures, and experimental variation in the degree of AI assistance.

## 6. Conclusions

Among Chinese university students with AI-writing experience, broader positive academic emotions carried the larger indirect statistical association between AI-assisted writing experience and general academic self-efficacy, while broader negative academic emotions carried a smaller association. The pattern remained after emotional-motivation items were removed from the predictor. A statistical suppression pattern appeared when positive emotions were controlled, but its psychological source is unknown. The larger positive indirect association should be interpreted cautiously given the limited discriminant validity between positive emotions and self-efficacy and the concurrent self-report design.

The results show that reported GenAI-assisted writing experience covaries with broader capability beliefs and academic emotions; they do not establish effects of AI use on emotions, self-efficacy, intelligence, writing performance, or academic achievement, or demonstrate a causal emotional mechanism. Stronger longitudinal, experimental, performance-based, process-tracing, and independently validated measurement evidence is required before concluding that GenAI-assisted writing changes students’ general academic capability or cognitive functioning.

## Figures and Tables

**Table 1:** Demographic characteristics of study participants (N = 1128).

Variable	Category	n	%
Gender	Male	526	46.6
	Female	602	53.4
University type	Double First-Class universities	549	48.7
	Other regular undergraduate universities	579	51.3
Academic level	First-year undergraduate	142	12.6
	Second-year undergraduate	244	21.6
	Third-year undergraduate	250	22.2
	Fourth-year undergraduate	214	19.0
	Graduate student	278	24.6
Discipline	Humanities and social sciences	660	58.5
	STEM and medical sciences	468	41.5
AI use frequency	Rarely	97	8.6
	Occasionally	319	28.3
	Frequently	460	40.8
	Always	252	22.3
Academic rank	Top 20%	340	30.1
	20–40%	379	33.6
	40–60%	322	28.5
	60–80%	62	5.5
	Bottom 20%	25	2.2

Note: Humanities and social sciences include philosophy, economics, law, education, literature, history, management, and arts. STEM and medical sciences include natural sciences, engineering, agriculture, medicine, and military science.

**Table 2:** Measurement properties of study variables.

Construct	Source	Items	Dimensions	α	CR	AVE	Factor Loading Range
AI-assisted writing experience	Khojasteh et al. (2025)	28	5	0.915	0.879 ^a^	0.612 ^a^	0.304–0.909 ^a^
Positive academic emotions	Bieleke et al. (2021)	24	3	0.952	0.947 ^b^	0.857 ^b^	0.906–0.950 ^b^
Negative academic emotions	Bieleke et al. (2021)	40	5	0.973	0.943 ^b^	0.769 ^b^	0.788–0.933 ^b^
Academic self-efficacy	Liang (2000)	22	2	0.915	0.909 ^b^	0.713 ^b^	0.800–0.871 ^b^

Note: ^a^ Based on five AI subscale-mean parcels derived from the exploratory, sample-specific reduced 28-item form in the final four-construct CFA; the item-level five-factor CFA is reported in Section 3.2.1. ^b^ Based on dimension-preserving parcels in the same final CFA. α = Cronbach’s alpha; CR = composite reliability; AVE = average variance extracted.

**Table 3:** Fornell–Larcker discriminant validity matrix (parcel-level four-factor model).

Construct	AI Writing	Positive Emotions	Negative Emotions	Self-Efficacy
AI-assisted writing	0.782			
Positive emotions	0.676	0.926		
Negative emotions	−0.434	−0.660	0.877	
Academic self-efficacy	0.741	0.942	−0.642	0.845

Note: diagonal values represent the square root of AVE for each construct. Off-diagonal values are standardized latent correlations estimated in the same parcel-level four-factor CFA.

**Table 4:** HTMT discriminant validity results.

Construct Pair	HTMT	95% Bootstrap CI
AI writing–positive emotions	0.693	[0.627, 0.750]
AI writing–negative emotions	0.418	[0.359, 0.480]
AI writing–self-efficacy	0.752	[0.693, 0.810]
Positive emotions–negative emotions	0.656	[0.604, 0.708]
Positive emotions–self-efficacy	0.946	[0.920, 0.967]
Negative emotions–self-efficacy	0.641	[0.586, 0.693]

**Table 5:** Descriptive statistics and Pearson correlation matrix (N = 1128).

Variable	M	SD	1	2	3	4
1. AI-assisted writing experience	3.818	0.532	—			
2. Positive academic emotions	3.841	0.656	0.607 ***	—		
3. Negative academic emotions	2.424	0.815	−0.329 ***	−0.624 ***	—	
4. Academic self-efficacy	3.746	0.582	0.643 ***	0.877 ***	−0.596 ***	—

Note: *** *p* < .001. All variables are scale means ranging from 1 to 5.

**Table 6:** Parallel pathway analysis results (N = 1128; bootstrap resamples = 5000).

Path	B (β)	*p*	95% CI
Total association (c): AI writing → Self-efficacy	0.733 (0.670)	<.001	—
a_1_: AI writing → Positive emotions	0.778 (0.631)	<.001	—
a_2_: AI writing → Negative emotions	−0.457 (−0.298)	<.001	—
b_1_: Positive emotions → Self-efficacy	0.606 (0.683)	<.001	—
b_2_: Negative emotions → Self-efficacy	−0.073 (−0.102)	<.001	—
Direct association (c′): AI writing → Self-efficacy	0.229 (0.209)	<.001	—
Indirect associations			
**Via positive emotions (a_1_ × b_1_)**	0.471 (0.431)	—	[0.409, 0.531]
Via negative emotions (a_2_ × b_2_)	0.033 (0.030)	—	[0.018, 0.052]
Total indirect association	0.504 (0.461)	—	[0.447, 0.559]
Contrast (positive − negative)	0.438 (0.400)	—	[0.365, 0.506]

Note: The model controls for academic level, university type, AI-use frequency, and academic-rank dummy variables (top 20% reference). Entries are unstandardized B with fully standardized β in parentheses. CIs are percentile case-bootstrap 95% confidence intervals on the unstandardized scale (5000 resamples; seed = 20,260,721). Total R^2^ = 0.799.

## Data Availability

The raw data supporting the conclusions of this article will be made available by the authors upon request.

## Supplementary Materials

The following supporting information can be downloaded at: https://www.mdpi.com/article/10.3390/jintelligence14090221/s1, Section S1: Scoring and Measurement Sensitivity; Section S2: Outcome-Dimensional Sensitivity; Table S1: Outcome-dimensional sensitivity estimates; Section S3: Relative Weight Analysis; Table S2: Relative weight analysis across AI-writing dimensions; Section S4: Robustness Checks; Section S5: Enhanced Covariate Sensitivity; Section S6: Data-Quality Screening Details.
