# Supplementary material for: Generative AI-Assisted Academic Writing Experience and University Students’ Academic Self-Efficacy: Parallel Indirect Associations Through Academic Emotions
Source: J Intell. 2026 Sep 15;14(9):221. doi: 10.3390/jintelligence14090221 (PMC13608356; doi:10.3390/jintelligence14090221)
Supplement: Supplementary file 1 [file jintelligence-14-00221-s001.zip › jintelligence-4527305-supplementary.pdf]

Supplementary Material

Generative AI-Assisted Academic Writing Experience and University Students' Academic Self-Efficacy: Parallel Indirect Associations Through Academic Emotions

Journal: Journal of Intelligence

This anonymous supplement reports sensitivity, dimensional, and robustness analyses supporting the focal parallel pathway model. Unless otherwise noted, all analyses use the same 1,128 complete cases as the main manuscript.

S1. Scoring and Measurement Sensitivity

The AI-assisted writing composite was calculated as the mean of the exploratory, sample-specific 28-item solution after excluding the technical-problems and originality-concern items identified during item analysis in the full sample. The reduced form should not be treated as independently validated.

The final self-efficacy parceling respected the confirmed two-domain structure: items 1–11 assessed learning-ability efficacy and items 12–22 assessed learning-behavior efficacy, with two balanced parcels formed within each domain. The positive-emotion–self-efficacy HTMT estimate was .946; the 5,000-resample percentile interval was [.920, .967]. An item-level sensitivity estimate was .926, 95% CI [.900, .948]. The four-factor model, $\chi^2(113) = 945.03$, CFI = .955, TLI = .946, RMSEA = .081, fit better than the model merging positive emotions and efficacy, $\chi^2(116) = 1152.33$, CFI = .944, TLI = .935, RMSEA = .089; $\Delta\chi^2(3) = 207.30$, $p < .001$, ΔCFI = −.011. Thus, the formal comparison favors separate factors, but HTMT and the small CFI decrement indicate limited discriminant validity and require cautious interpretation.

As a post hoc internal-stability check, the 28-item five-factor AI-AWQ model was estimated in two random halves (n = 564 each; seed = 20,260,726). Fit was $\chi^2(340) = 842.25$, CFI = .918, TLI = .909, RMSEA = .051 in half 1 and $\chi^2(340) = 860.55$, CFI = .906, TLI = .896, RMSEA = .052 in half 2. Both models converged. Because item deletion was informed by the full sample, this is an internal stability check rather than independent validation.

S2. Outcome-Dimensional Sensitivity

The parallel pathway model was re-estimated separately for learning-ability and learning-behavior self-efficacy with the same covariates and 5,000 bootstrap resamples.

Table S1. Outcome-dimensional sensitivity estimates.

Outcome	Positive-emotion indirect association	Negative-emotion indirect association	Direct association
Learning ability efficacy	.519 [.448, .589]	.035 [.016, .056]	.232
Learning behavior efficacy	.423 [.361, .484]	.032 [.016, .050]	.225

Note. Values in brackets are 95% percentile case-bootstrap confidence intervals. Seeds were 20,260,723 for learning ability and 20,260,724 for learning behavior (5,000 resamples each).

S3. Relative Weight Analysis

Relative weight analysis decomposed explained variance across the five correlated AI-writing dimensions. Perceived effectiveness and emotional motivation were the two largest contributors across the three outcomes.

Table S2. Relative weight analysis across AI-writing dimensions.

Dimension	Academic self-efficacy RW (%)	Positive emotions RW (%)	Negative emotions RW (%)
Emotional motivation	.132 (27.7%)	.121 (28.8%)	.071 (35.7%)
Perceived effectiveness	.151 (31.7%)	.127 (30.2%)	.056 (28.3%)
Feedback improvement	.099 (20.8%)	.086 (20.6%)	.035 (17.8%)
Writing process	.079 (16.6%)	.072 (17.1%)	.026 (13.3%)
Ethical considerations	.015 (3.2%)	.014 (3.3%)	.010 (4.9%)
Total R^2	.476	.420	.198

Note. RW = relative weight (raw). Percentages represent proportional contribution to total R^2.

S4. Robustness Checks

To reduce direct content overlap between the predictor and the proposed emotional pathways, a revised AI-writing predictor was computed as the equal-weight mean of the 24 retained items from four subscales—writing process, feedback improvement, perceived effectiveness, and ethical considerations—after excluding all four emotional-motivation items. The main pattern remained: total association = .700, direct association = .213, positive-emotion indirect association = .457, 95% CI [.391, .517], and negative-emotion indirect association = .030, 95% CI [.016, .047]. The AI coefficient predicting residual negative emotion after positive emotion was controlled remained positive (B = .227, $p < .001$), reproducing the statistical suppression pattern. This analysis reduces explicit emotional content in the predictor but cannot eliminate common method variance or broader evaluative overlap. The model used 5,000 percentile case-bootstrap resamples with seed 20,260,722.

Without covariates, estimates remained close to those of the primary model: total association c = .704, a1 = .749, a2 = −.504, b1 = .625, b2 = −.069, direct association c′ = .201, positive indirect association = .468, 95% CI [.405, .526], and negative indirect association = .035,

95% CI [.018, .054]. All constituent regression paths were significant at $p < .001$. This model used 5,000 percentile case-bootstrap resamples with seed 20,260,727.

Across the non-emotional-predictor, no-covariate, and efficacy-domain models, broader positive academic emotions remained the dominant indirect pathway. The primary model used seed 20,260,721; the parcel-level HTMT analysis used seed 20,260,720; the item-level HTMT sensitivity used seed 20,260,725; and the no-covariate model used seed 20,260,727.

S5. Enhanced Covariate Sensitivity

As an enhanced sensitivity analysis, academic level and AI-use frequency were dummy-coded rather than entered as ordered numeric scores, and gender and broad discipline were added to the university-type and academic-rank controls used in the primary model. The pattern remained stable: total association = .726, direct association = .230, positive-emotion indirect association = .463, 95% CI [.397, .526], and negative-emotion indirect association = .032, 95% CI [.017, .051]. Confidence intervals were estimated using 5,000 case-bootstrap resamples with seed 20,260,726.

S6. Data-Quality Screening Details

The survey yielded 1,278 submissions, of which 1,128 (88.3%) were retained. Screening required a completion time of at least 300 seconds, a correct response to the instructed-response attention check, and the absence of invariant response patterns across the 116 administered substantive items, including the two AI-AWQ items that were subsequently excluded from scale scoring. Among retained cases, completion times ranged from 300 to 992 seconds (median = 435 seconds); all 1,128 passed the attention check, and none showed invariant responding across all substantive items. The archived analytic files do not preserve mutually exclusive counts for each reason among the 150 excluded submissions, so category-specific exclusion counts cannot be reconstructed and are not reported.
